# P-2175. Prospective study on the clinico-epidemiological characteristics, diagnostic approaches and correlation with autopsy findings of rabies encephalitis

**DOI:** 10.1093/ofid/ofae631.2329

**Published:** 2025-01-29

**Authors:** Madhav Mohata, Paramjeet Singh, Gursimran Kaur Mohi, Radha Kanta, Vikas Suri

**Affiliations:** All India Institute of Medical Sciences, New Delhi, New Delhi, Delhi, India; Post Graduate Institute of Medical Education and Research, Chandigarh, Chandigarh, Chandigarh, India; Post Graduate Institute of Medical Education and Research, Chandigarh, Chandigarh, Chandigarh, India; Post Graduate Institute of Medical Education and Research, Chandigarh, Chandigarh, Chandigarh, India; Post Graduate Institute of Medical Education and Research, Chandigarh, Chandigarh, Chandigarh, India

## Abstract

**Background:**

Rabies is the deadliest infectious disease, with a nearly 100% fatality. Due its fear, it is often overlooked, resulting in scarcity of comprehensive knowledge, most of which is derived from retrospective studies. This prospective study is aimed to narrow this knowledge gap.

**Table 1:**
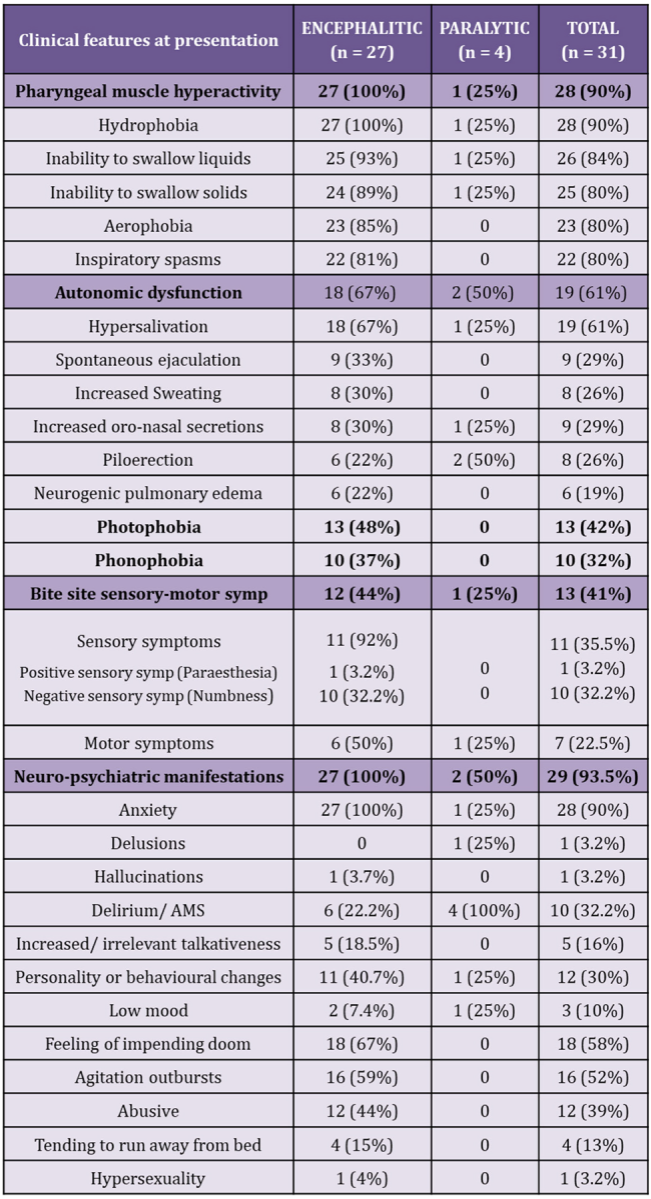
Clinical features

Table 1: Table summarizing the frequency of clinical features in both forms of rabies at the time of presentation

**Methods:**

This prospective observational study included 36 participants. Detailed clinical history and clinical examination was done, Milwaukee protocol was used for patient management. For diagnostics, CEMRI Brain was performed, and samples of CSF, saliva, serum, skin and brain biopsies were collected for various diagnostic tests. Autopsy was done for 8 patients.

Table 2 and Figure 1 & 2: Incubation period

**Figure:**
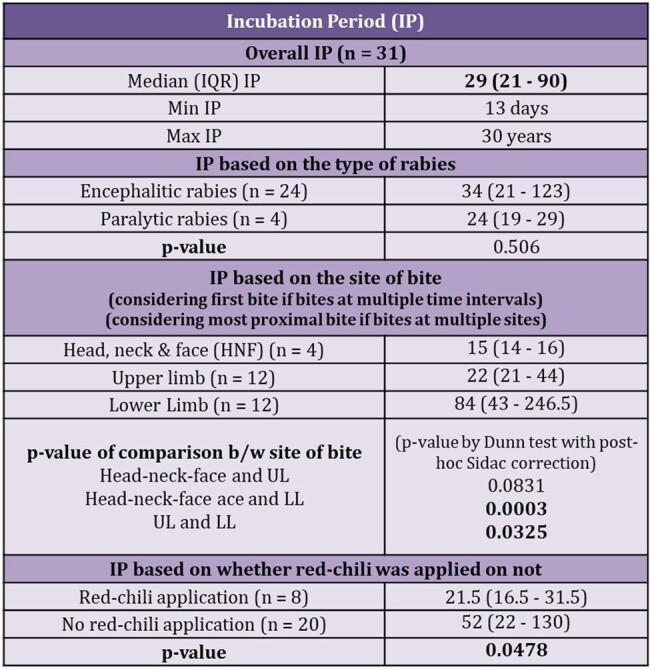

Table 2: Table representing various incubation periods, in median (IQR), and the respective p-values between 2 groups

Figure 1: Figure demonstrating statistically significant trend of shorter incubation period with proximity of site of bite to CNS. P-values are calculated using Dunn test with post-hoc Sidac correction

Figure 2: Box plot representing the difference between incubation period based on no application of red-chilli vs application of red-chilli, p-value (ranksum test) = 0.0478

**Results:**

The study unearthed numerous intriguing aspects of the disease. Despite presence of adequate infrastructure, only 48% initiated PEP, and a mere 26% received Rabies Immunoglobulin. Adherence to the vaccination schedule was subpar, with only 13.3% completing full course. The median incubation period was 29 days, with variations based upon the type of rabies, bite site, and irritant application. Autonomic dysfunction emerged as prominent manifestation, with variations in heart rate (90%), BP (90%), and temperature (81%); hypersalivation (61%), hyperhidrosis, spontaneous ejaculations (29%), and neurogenic pulmonary edema (19%) being the common features. MRI revealed dorsal brainstem (90%), peri-aqueductal grey (72.7%), basal ganglia (63.6%), and cervical spinal cord (72.2%) as most affected areas. Antemortem diagnosis showed the following positivity rates: nape of neck skin biopsy IHC (40%), saliva-PCR (23.8%), skin biopsy PCR (20%), and CSF-PCR (4.3%). Postmortem brain biopsy PCR demonstrated a 100% positivity. Autopsy findings revealed the following – negri bodies, perivascular lymphocytic infiltrate, neuronophagia and microglial formation or nodule formation in almost all areas of the brain with maximum in hippocampus, cortex, and brainstem.

Table 3 and 4: Diagnostic tests and MRI features

**Figure:**
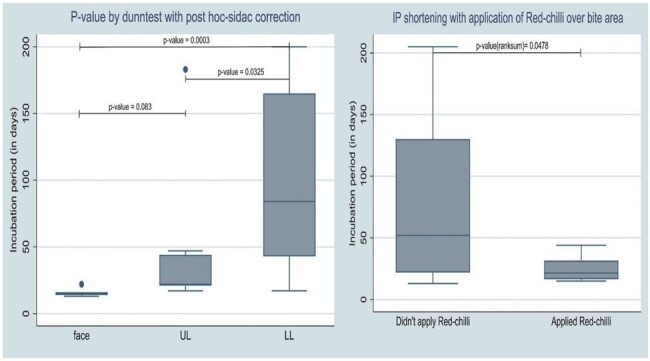

Table 3: Diagnostic performance of various tests

Table 4: Table summarizing the MRI findings in rabies patients

**Conclusion:**

This is the first of its kind prospective study, which studies in-depth the clinico-epidemilogic, neuroimaging and autopsy features and the performance of various diagnostic tests in rabies. The unique features of the study include – its prospective nature, prominent clinical features of dysautonomia, neuroimaging characteristics and their corelation with autopsy.

Figure 3, 4, 5 & 6: Histopathology and immunohistochemistry findings in autopsy

**Figure:**
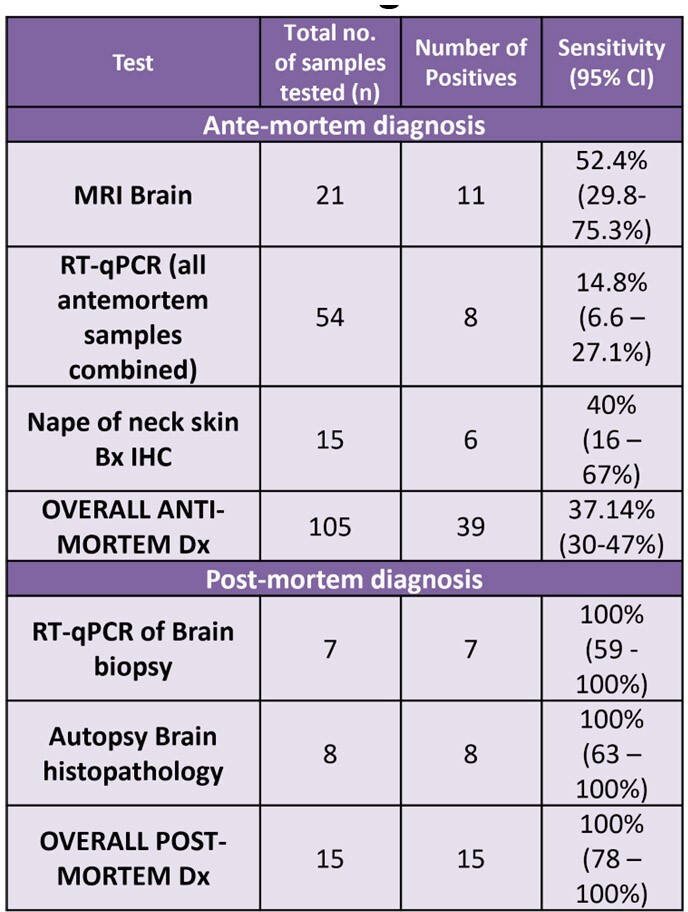

Figure 3: Perivascular lymphocytic cuffing

Figure 4: Negri bodies in the cortical neurons and purkinje cells of cerebellum

Figure 5: Microglial nodule and diffuse microglial proliferation

Figure 6: Immunohistochemistry demonstrating rabies virus antigen within the neurons seen as diffuse granular cytoplasmic positivity within the neurons, its axons and dendrites

**Disclosures:**

All Authors: No reported disclosures

